# Association of anaemia with *Helicobacter pylori* infection: a retrospective study

**DOI:** 10.1038/s41598-017-13955-3

**Published:** 2017-10-18

**Authors:** Mei-Yan Xu, Bing Cao, Bao-Shi Yuan, Jian Yin, Lan Liu, Qing-Bin Lu

**Affiliations:** 1 Department of Nutrition, Aerospace Center Hospital, Beijing, 100049 P. R. China; 2 Department of Laboratorial Science and Technology, School of Public Health, Peking University, Beijing, 100191 P. R. China; 3 Department of Health Management, Aerospace Center Hospital, Beijing, 100049 P. R. China; 4 Beijing Key Laboratory of Toxicological Research and Risk Assessment for Food Safety, Beijing, 100191 P. R. China

## Abstract

The role of Helicobacter pylori (*H*. *pylori*) infection in haematological system diseases is not well understood. We conducted this retrospective study to explore the association between *H*. *pylori* infection and anaemia in the Chinese population. This retrospective study was performed in Aerospace Center Hospital in Beijing. We derived the data from the registration system of the physical population between 2012–2016. Logistic regression models were used to explore the association between *H*. *pylori* infection and anaemia. Among 17,791 subjects, there were 7,804 (43.9%) subjects with *H*. *pylori* infection and 950 (5.3%) with anaemia. The prevalence of anaemia in the *H*. *pylori* (+) group was significantly higher than in the *H*. *pylori* (−) group after adjusting for age, sex, marriage, underlying diseases and body mass index. Compared to *H*. *pylori* (−), the OR of *H*. *pylori* (+) was 1.39 for moderate-to-severe anaemia and 1.05 for mild anaemia. The level of haemoglobin was lower in the *H*. *pylori* (+) group than in the *H*. *pylori* (−) group. This study indicates that *H*. *pylori* infection may be related to anaemia and haemoglobin level in the Chinese population.

## Introduction

Anaemia is a serious public health problem, and its prevalence remains unacceptably high in many regions^1–3^, affecting roughly a third of the world’s population^4^. Anaemia is usually associated with a variety of other diseases, such as malaria, schistosomiasis, and chronic kidney disease^5^.

*Helicobacter pylori* (*H*. *pylori*) is a spiral shaped pathogenic bacterium found on the human gastric mucosa^6^, and its prevalence remains high in many regions of the world^7^. The prevalence of *H*. *pylori* infection was reported to range from 8.7% to 85.5%^8^, which increased with age and varies widely by geographic area, race, ethnicity and socio-economic status^9^. *H*. *pylori* infection plays a major role in the incidence of chronic gastritis^10^, but the involvement of its infection on haematological system diseases is not well understood.

*H*. *pylori* gastric colonization generally persists for decades and requires the continuous supplementation of nutrients essential for bacterial growth^2^. Rostami-Nejad *et al*. indicated that *H*. *pylori* was associated with iron deficiency anaemia even in celiac disease patients, which was strongly evidence based but weakly reflected in practice^11^. A range of evidence from epidemiological and clinical studies supports an association between anaemia and *H*. *pylori* infection. However, reports from different areas and countries are not consistent regarding this association between anaemia and *H*. *pylori* infection^1^ and the underlying mechanisms remain unclear.

As observed in one meta-analysis^1^, which included most of the previous studies on anaemia and *H*. *pylori*, the past studies were imperfect with notable shortcomings. First, most previous international researches on *H*. *pylori* infection and anaemia was concentrated on women and children, especially pregnant women, rather than the general population, with relatively small sample sizes. Most also did not consider the severity and type of the anaemia. For studies with large sample studies, the information was often inadequate to account for potential confounding factors. To our knowledge, no published studies with large sample sizes have explored the association between *H*. *pylori* and anaemia in the Chinese adult population. Therefore, we conducted this retrospective study using data from the registration system of the physical population to explore the association between *H*. *pylori* infection and anaemia in the Chinese population.

## Methods

### Study design and participants

This retrospective study was performed at the Aerospace Center Hospital in Beijing, China. The subjects in this study were a healthy population who underwent health examinations at this hospital between 2012 and 2016. Any subjects without results of *H*. *pylori* infection status (anti-*H*. *pylori* IgG and IgM) were excluded. Subjects without data for mean cell volume (MCV) and haemoglobin, red blood cell (RBC), mean cell haemoglobin (MCH), and mean cell haemoglobin concentration (MCHC) were also excluded

A chart review of all recruited subjects was performed to collect information related to demographic characteristics, underlying diseases (diabetes, hypertension and coronary heart disease), and the results of haematological parameters (MCV, haemoglobin, RBC, MCH and MCHC). The data of the height and weight of the subjects were also collected. Body mass index (BMI) was calculated by the weight (kg) divided by the square of height (m). BMI groups were defined as obese with BMI ≥ 28 kg/m^2^, overweight with 24 ≤ BMI < 28 kg/m^2^, normal with 18.5 ≤ BMI < 24 kg/m^2^, underweight with BMI < 18.5 kg/m^2^, and non-obese was BMI < 28 kg/m^2^, according to the criteria of weight for adults in the Health industry standard of China (WS/T 428–2013).

*H*. *pylori* status was estimated by enzyme linked immunosorbent assay using anti-*H*. *pylori* IgG and IgM. This study was performed with the approval of the Ethical Committees of Aerospace Center Hospital, and all methods and protocols were carried out in accordance with the approved guidelines and regulations. At recruitment, written informed consent was obtained from all subjects.

### Outcomes

The key outcome (anaemia) was measured objectively using WHO haemoglobin cutoffs^12^. We defined anaemia as a haemoglobin level no more than 130 g/L for men and no more than 120 g/L for women according to WHO sex-based criteria. Patients with anaemia were further divided into two groups according to the severity of anaemia: mild anaemia (110 g/L ≤ hemoglobin < 119 g/L for women and 110 ≤ hemoglobin < 129 g/L for men) and moderate-severe anaemia (hemoglobin < 110 g/L for men and women). Patients with anaemia were also divided into four groups according to the type of anaemia: normocytic anaemia (NA, 80 ≤ MCV ≤ 100 pg), simple small cell anaemia (SCA, MCV < 100 pg and 320 ≤ MCHC ≤ 360 fl), large cell anaemia (LCA, MCV > 100 pg), and microcytic hypochromic anaemia (MHA, MCV < 100 pg and MCHC < 320 fl)^13^.

### Statistical analysis

Descriptive statistics were performed, with continuous variables summarized as the mean and standard deviation (SD), and categorical variables summarized as frequencies and proportions. Statistical significance between various groups was tested using the χ^2^ test for categorical variables and independent t-test for continuous variables. Generalized linear models were used to explore the association between *H*. *pylori* infection and haemoglobin values. Logistic regression models were used to explore the association between *H*. *pylori* infection and anaemia. Ordinal logit models were used to explore the association between *H*. *pylori* infection and differences in severity or type of anaemia. The variables of age, sex, diabetes, hypertension, coronary heart disease and BMI were adjusted for the above models. As anaemia prevalence is quite different for males or females, we also analysed the effect of sex using all the above models. Odds ratios (ORs) and their 95% confidence intervals (CIs) were estimated. A two-sided *P* < 0.05 was deemed statistically significant. All analyses were performed using Stata 12.0 (Stata Corp LP, College Station, TX, USA).

## Results

### Description of subjects & *H*. *pylori* and anaemia distribution

A total of 17,791 subjects were recruited from the hospital for this study. Subjects had a mean age (SD) of 46 (18) years, and 5,844 (32.5%) were female. The overall prevalence of underlying diseases was 30.2% (5,374/12,417), including 11.7% with diabetes, 25.1% with hypertension and 4.6% with coronary heart disease. As for BMI, 27.3% of the subjects were overweight and 12.6% were obese. All basic characteristics of subjects included in the study are shown in Table 1.

**Table 1:** The basic characteristics of the subjects and the prevalence of *H*. *pylori* infection and anemia in the study

**Variable**	**Total (N = 17791)**	***H***. ***pylori***			***Anemia***		
		**Positive (n = 7804)**	**Negative (n = 9987)**	***P*** **value**	**Yes (n = 2250)**	**No (n = 15541)**	***P*** **value**
Age, year, Mean ± SD	45 ± 18	46 ± 18	45 ± 18	<0.001	54 ± 20	45 ± 18	<0.001
≤45	10606 (59.6)	4425 (41.2)	6181 (58.3)	<0.001	424 (4.0)	10182 (96.0)	<0.001
45~60	2847 (16.0)	1352 (47.5)	1495 (52.5)		143 (5.0)	2704 (95.0)	
>60	4338 (24.4)	2027 (46.7)	2311 (53.3)		383 (8.8)	3955 (91.2)	
Sex, female (%)	5844 (32.5)	2325 (39.8)	3519 (60.2)	<0.001	649 (11.0)	5195 (88.9)	<0.001
Underlying diseases	5374 (30.2)	2460 (45.8)	2914 (54.2)	0.001	345 (6.4)	5029 (93.6)	<0.001
Diabetes	2078 (11.7)	927 (44.6)	1151 (55.4)	0.466	140 (6.7)	1938 (93.3)	0.003
Hypertension	4467 (25.1)	2068 (46.3)	2399 (53.7)	<0.001	298 (6.7)	4169 (93.3)	<0.001
Coronary heart disease	813 (4.6)	348 (42.8)	465 (57.2)	0.533	96 (11.8)	717 (88.2)	<0.001
LDL > 3.1 mmol/L	4673 (26.3)	2177 (46.6)	2496 (53.4)	<0.001	136 (2.9)	4537 (97.1)	<0.001
HDL<0.83 mmol/L	5286 (29.1)	2436 (46.1)	2850 (53.1)	<0.001	213 (4.0)	5073 (96.0)	<0.001
TG > 1.71 mmol/L	4512 (25.4)	2039 (45.2)	2473 (54.8)	0.038	121 (2.7)	4391 (97.3)	<0.001
TC > 5.7 mmol/L	3253 (18.3)	1463 (45.0)	1790 (55.0)	0.159	111 (11.7)	3142 (88.3)	<0.001
Hemoglobin, g/L	147 ± 15	147 ± 14	147 ± 15	0.053	114 ± 12	149 ± 13	<0.001
Male	154 ± 11	154 ± 11	155 ± 11	<0.001	122 ± 10	154 ± 11	<0.001
Female	132 ± 11	132 ± 11	133 ± 11	0.393	111 ± 10	135 ± 8	<0.001
BMI, kg/m^2^	24.1 ± 3.5	24.3 ± 3.5	24.0 ± 3.4	<0.001	22.9 ± 3.5	24.2 ± 3.4	<0.001
Underweight	498 (3.4)	197 (39.6)	301 (60.4)	0.134	54 (10.8)	444 (89.2)	<0.001
Normal	7012 (47.3)	2929 (41.8)	4083 (58.2)		454 (6.5)	6558 (93.5)	
Overweight	5453 (27.3)	2503 (45.9)	2950 (54.1)		197 (3.6)	5256 (96.4)	
Obesity	1869 (12.6)	875 (46.8)	994 (53.2)		65 (3.5)	1804 (96.5)	

SD, standard deviation; BMI, body mass index.

Among all subjects, there were 7,804 (43.9%) subjects with *H*. *pylori* infection and 950 (5.3%) with anaemia. The subjects with older age, hypertension and higher BMI value were demonstrated to have higher prevalence of *H*. *pylori* infection (all P < 0.05). There were no significant differences in the prevalence of *H*. *pylori* for diabetes, coronary heart disease or BMI groups (all P > 0.05). Significant differences in the rates of anaemia were found for the variables of age, sex, underlying diseases and BMI (all P < 0.05).

### Association between *H*. *pylori* and the severity of anaemia

The prevalence of anaemia was 5.5% (428/7,804) in the subjects with *H*. *pylori* infection compared to 5.2% (522/9,987) in uninfected individuals. The prevalence of anaemia in the *H*. *pylori* (+) group was significantly higher than in the *H*. *pylori* (−) group after adjusting for age, sex, marriage, underlying diseases and BMI (OR = 1.19; 95% CI: 1.03, 1.39; P = 0.021), as shown in Table 2. When classified by sex, the anaemia rate in the *H*. *pylori* (+) group was significantly higher than in the *H*. *pylori* (−) group for the female subjects (OR = 1.25; 95% CI: 1.04, 1.51; P = 0.019), but no significant association was detected for the male subjects (OR = 1.09; 95% CI: 0.84, 1.40; P = 0.530).

**Table 2:** The association between *H*. *pylori* infection and the severity of anemia in the study.

**Factor**	**n (%)**	***H***. ***pylori*** (+)	***H***. ***pylori*** **(−)**	**Crude**			**Adjusted** ^a^		
				**OR**	**95%CI**	**P**	**OR**	**95%CI**	**P**
**Model 1 in all the subjects**									
Normal	16841 (94.8)	7376 (94.5)	9465 (94.8)	1			1		
Any anemia	950 (5.3)	428 (5.5)	522 (5.2)	1.05	0.92, 1.12	0.450	1.19	1.03, 1.39	0.021
Mild anemia	736 (4.1)	325(4.2)	411 (4.1)	1.02	0.87, 1.18	0.850	1.05	0.90, 1.22	0.520
Moderate-to-severe anemia	214 (1.2)	103(1.3)	111 (1.1)	1.19	0.91, 1.56	0.210	1.39	1.06, 1.82	0.019
**Model 2 in the male subjects**									
No anemia	11646 (97.5)	5334 (97.4)	6312 (97.6)	1			1		
Any anemia	301 (2.5)	145 (2.7)	156 (2.4)	1.11	0.88, 1.38	0.420	1.09	0.84, 1.40	0.530
Mild anemia	274 (2.3)	128 (2.3)	146 (2.3)	1.04	0.82, 1.32	0.760	2.01	0.92, 4.40	0.890
Moderate-to-severe anemia	27 (0.2)	17 (0.3)	10 (0.1)	1.02	0.78, 1.33	0.080	2.41	0.91, 6.34	0.076
**Model 3 in the female subjects**									
No anemia	5195 (88.9)	2042 (87.8)	3153 (89.6)	1			1		
Any anemia	649 (11.1)	283 (12.2)	366 (10.4)	1.19	1.01, 1.41	0.035	1.25	1.04, 1.51	0.019
Mild anemia	462 (7.9)	197 (8.5)	265 (7.5)	1.15	0.95, 1.40	0.160	1.21	0.98, 1.51	0.079
Moderate-to-severe anemia	187 (3.2)	86 (3.7)	101 (2.9)	1.32	0.98, 1.76	0.067	1.35	0.96, 1.89	0.083

^a^Adjusted OR and 95%CI were calculated by adjusting for the potential confounders, including age, sex, underlying diseases and BMI.

The ordinal logit model was used to analyse the association between *H*. *pylori* infection and the severity of anaemia. Compared to *H*. *pylori* (−), the OR of *H*. *pylori* (+) was 1.39 (95% CI: 1.06, 1.54; P = 0.019) for moderate-to-severe anaemia and 1.05 (95% CI: 0.90, 1.22; P = 0.520) for mild anaemia after adjusting for potential confounders (Table 2). Significant associations between *H*. *pylori* infection and the severity of anaemia were also not found in the male and female models.

### Associations between *H*. *pylori* infection and type of anaemia

The association between *H*. *pylori* infection and the type of anaemia was analysed in this study. Haematological parameters for both the *H*. *pylori* (+) group and the *H*. *pylori* (−) group are summarized in Table 3. The levels of RBC, MCV, MCH, MCHC had significant differences between *H*. *pylori* (+) and *H*. *pylori* (−) groups (all P < 0.05). However, these differences were small and likely lack biological significance. The association between *H*. *pylori* infection and the type of anaemia were analysed using a multinomial logistic regression model (Table 4). Significant differences were not detected between *H*. *pylori* infection and different anaemia types in the all subjects model, female model and male model. When adjusted for potential confounders, including age, sex, underlying diseases and BMI, a significant association was found between *H*. *pylori* infection and NA (OR = 1.25; 95% CI: 1.04, 1.49; P = 0.017). This association was also found in the female model (OR = 1.28; 95% CI: 1.02, 1.60; P = 0.035) after adjusting for the above factors.

**Table 3:** The association between *H*. *pylori* and hematological parameters in the study.

**Variables**	**Overall (N = 17791)**	***H***. ***pylori*** **(+)**	***H***. ***pylori*** (−)	**P**
RBC (×10^12^/L)	4.89 ± 0.49	4.89 ± 0.48	4.89 ± 0.49	0.005
MCH (pg)	30.14 ± 1.82	30.17 ± 1.82	30.11 ± 1.82	0.019
<27	550 (3.2)	247 (3.2)	303 (5.1)	0.042
27~34	16636 (96.0)	7320 (95.9)	9316 (96.0)	
>34	146 (0.8)	63 (0.8)	83 (0.9)	
MCHC (g/L)	335.2 ± 8.8	335.1 ± 8.9	335.4 ± 8.7	0.028
<320	578 (3.3)	274 (3.6)	304 (3.1)	0.015
320~360	16742 (96.6)	7354 (96.4)	9388 (96.8)	
>360	12 (0.1)	2 (0.0)	10 (0.1)	
MCV (fl)	89.9 ± 4.9	90.0 ± 4.9	89.8 ± 4.9	0.002
<80	377 (2.2)	173 (2.3)	204 (2.1)	0.052
80~100	16669 (96.2)	7341 (96.2)	9358 (96.5)	
>100	256 (1.5)	116 (1.5)	140 (1.4)

**Table 4:** The association between *H*. *pylori* infection and the type of anemia in the study.

**Type**	**Total**	***H***. ***pylori*** (+)	***H***. ***pylori*** **(−)**	**Crude**			**Adjusted** ^a^		
	**n (%)**			**OR**	**95%CI**	**P**	**OR**	**95%CI**	**P**
**Model 1 in all the subjects**									
No anemia	16841 (94.8)	7376 (94.5)	9465 (94.8)	1			1		
NA	644 (3.6)	293 (3.7)	352 (3.5)	1.06	0.91, 1.25	0.44	1.25	1.04, 1.49	0.017
LCA	50 (0.3)	23 (0.3)	27 (0.3)	1.09	0.63, 1.91	0.75	0.89	0.49, 1.63	0.71
SCA	47 (0.3)	17 (0.2)	30 (0.3)	0.73	0.40, 1.32	0.29	1.10	0.56, 2.14	0.78
MHA	209 (1.2)	96 (1.2)	103 (1.3)	1.09	0.83, 1.43	0.54	1.18	0.87, 1.62	0.29
**Model 2 in the male subjects**									
No anemia	11646 (97.5)	5334 (97.4)	6312 (97.6)	1			1		
NA	223 (1.9)	109 (2.0)	114 (1.8)	1.13	0.87, 1.48	0.36	1.17	0.87, 1.56	0.29
LCA	37 (0.3)	16 (0.3)	21 (0.3)	0.90	0.47, 1.73	0.76	0.79	0.39, 1.59	0.51
SCA	8 (0.1)	3 (0.1)	5 (0.1)	0.71	0.17, 1.97	0.64	0.85	0.19, 3.79	0.83
MHA	33 (0.3)	17 (0.3)	16 (0.3)	1.26	0.64, 2.49	0.51	1.07	0.50, 2.27	0.87
**Model 3 in the female subjects**									
No anemia	5195 (88.9)	2042 (87.8)	3153 (89.6)	1			1		
NA	421 (7.2)	183 (7.9)	238 (6.8)	1.19	0.97, 1.45	0.094	1.28	1.02, 1.60	0.035
LCA	13 (0.2)	7 (0.3)	6 (0.2)	1.80	0.61, 5.37	0.29	1.26	0.38, 4.15	0.71
SCA	39 (0.7)	14 (0.6)	25 (0.7)	0.87	0.45, 1.67	0.66	1.18	0.56, 2.49	0.66
MHA	176 (3.0)	79 (3.4)	97 (2.8)	1.26	0.93, 1.70	0.14	1.22	0.87, 1.73	0.25

^a^Adjusted OR and 95%CI were calculated by adjusting for the potential confounders, including age, sex, underlying diseases and BMI.

NA, normocytic anemia; SCA, simple small cell anemia; LCA, large cell anemia; MHA, microcytic hypochromic anemia.

### Association between *H*. *pylori* infection and the level of haemoglobin

The association between *H*. *pylori* infection and the level of haemoglobin were analysed using the generalized linear model in Table 5. When including all subjects, the haemoglobin level was lower in *H*. *pylori* (+) subjects than in *H*. *pylori* (−) subjects after adjusting for potential confounders (β = −0.63; 95% CI: −0.97, −0.28; P < 0.001). This finding was also observed for the male subjects group (β = −0.61; 95% CI: −1.00, −0.22; P = 0.002).

**Table 5:** Association between *H*. *pylori* and the level of hemoglobin.

Variable	Crude			Adjusted^a^		
	*β*	95% CI	P value	*β*	95% CI	P value
**Model 1 in all the subjects**						
*H. pylori* (+)	0.45	-0.005, 0.89	0.052–0.63	-0.63	-0.97, -0.28	<0.001
**Model 2 in the male subjects**						
*H. pylori* (+)	-0.93	-1.32, -0.53	<0.001	-0.61	-1.00, -0.22	0.002
**Model 3 in the female subjects**						
*H. pylori* (+)	-0.26	-0.84, 0.33	0.390	-0.54	-1.19, 0.12	0.110

^a^Adjusted *β* and 95%CI were calculated by adjusting for the potential confounders, including age, sex, underlying diseases and BMI.

## Discussion

In this retrospective study with a large sample size, we evaluated the relationship between *H*. *pylori* infection and anaemia using 17,791 subjects from the health examination population in Beijing. Subjects with *H*. *pylori* infection may have a higher possibility of anaemia and a lower level of haemoglobin.

The prevalence of *H*. *pylori* infection and anaemia was 43.9% and 5.5%, respectively, which were lower than those cited by other studies conducted on the Chinese adult population^14,15^. The prevalence of anaemia was 9.7% in the Chinese rural population^16^ and 9.7% in the urban population on in the 2010–2012 National Nutrition and Health Survey^15^. Another study showed that the prevalence of anaemia in urban community dwelling elderly population was 16.3% in men and 13.7% in women, which increased significantly with age^17^. This may be due to study populations from relatively developed areas in China. The prevalence of iron deficiency anaemia in Chinese population was 0.37% in the year of 2008 and 0.17% for male and 0.20% for female^18^. As we did not obtain the data on serum iron, serum ferritin and serum transferrin saturation, etc, we cannot calculate the prevalence of iron deficiency anaemia in our study. However, MHA may represent the prevalence of iron deficiency anaemia, which was higher in our study than the above.

The association between *H*. *pylori* infection and anaemia has been explored by previous epidemiological studies in different settings^9,15,19–23^. Anaemia is considered as a complication of *H*. *pylori* infection^24^. We found that the subjects with *H*. *pylori* infection had higher prevalence of anaemia, especially in female subjects. A meta-analysis found a borderline significant and weak positive association between *H*. *pylori* infection and anaemia with a pooled OR of 1.15 (95% CI: 1.00, 1.32)^1^. Moreover, that study also indicated that the magnitude of this association was stronger when the analysis was limited to studies that adjusted for confounders, which is consistent with our study.

There are several candidate anaemiagenic mechanisms that might explain the association between *H*. *pylori* infection and anaemia. The most plausible mechanism is gastrointestinal blood loss due to *H*. *pylori*-induced gastritis or duodenitis^25^. Another possibility is that the *H*. *pylori* bacterial sequestration of free iron affects iron transporter molecules, thereby inhibiting free iron absorption and causing food cobalamin malabsorption^26,27^. In addition, *H*. *pylori* gastric colonization requires continuous supplementation of nutrients essential for bacterial growth and can use the host’s own iron stores^28^. Severe anaemia was related to *H*. *pylori* infection in one case report on school age children, which recommends screening for *H*. *pylori* infection and appropriate treatment for severe iron-deficiency anaemia^29^. However, we did not find a significant association between *H*. *pylori* infection and severity of anaemia in this study. This may be due to the characteristics of our subjects, which had a selection bias.

Our analysis also revealed significant differences in RBC, MCV, MCH, and MCHC between *H*. *pylori* (+) and *H*. *pylori* (−) groups, but these changes are unlikely to be biologically significant. A health examination of 2,398 healthy subjects and a clinical study also indicated similar results concerning MCV, MCH, and MCHC^30,31^. Moreover, a large household controlled therapeutic trial among children with iron deficiency suggested that *H*. *pylori* infection might play a causal role in haematological outcomes^32^. In this study, we also analysed the associations between *H*. *pylori* infection and different types of anaemia. To our knowledge, this was the first to explore an association between *H*. *pylori* infection and anaemia type. After adjusting for potential confounders, subjects with *H*. *pylori* infection had higher prevalence of NA, and this association was also found in the female subjects. This finding requires further clinical or molecular studies to elucidate the mechanisms underlying the association between *H*. *pylori* infection and different types of anaemia.

We observed a lower haemoglobin level in the positive *H*. *pylori* infection group for male subjects. However, this association was not found in all subjects, which is similar with previous research from Taiwan^33^. Another study indicated that haemoglobin levels had statistical significance among no infection, recent infection, long-term infection and past infection subjects. That study concluded that a lower haemoglobin level was related to the presence of gastric atrophy and older age rather than the presence of *H*. *pylori* infection itself^30^. A community-based study of Arabs found a significantly lower haemoglobin level in children aged 6–9 years who were infected with *H*. *pylori* compared with their uninfected peers^34^. A meta-analysis of randomized control trials of *H*. *pylori* eradication has indicated that eradication can increase haemoglobin levels^35^. In addition, it was reported that levels of haemoglobin, serum iron and ferritin are improved following the treatment of *H*. *pylori* infection. Based on the above results, we believe that an association between *H*. *pylori* infection and haemoglobin level exists. However, the underlying reason for this still requires further research, especially for male subjects.

Our findings should lead to careful consideration of appropriate interventions aimed at eradication of *H*. *pylori*, not only for the possibility to improve anaemia status but also to provide protection against underlying diseases (e.g., hypertension), although this will require much effort.

Our study has several limitations. First, we cannot confirm the actual causality between *H*. *pylori* infection and anaemia as this is an observational study. Second, all subjects were from the health examination population at Aerospace Center Hospital in Beijing, which leads to selection bias. The third limitation is that serological testing for the presence of anti-*H*. *pylori* IgG and IgM does not indicate a current infection and only shows exposure to these bacteria, which may have biased the detection of *H*. *pylori* infection. Moreover, we did not obtain iron status laboratory test results, so we cannot give the detailed information on the subjects with iron deficiency anaemia. Finally, the ratio of males to females was high due to the characteristics of the unit from which the subjects came. The above limitations might have biased our results.

## Conclusion

This study indicates that *H*. *pylori* infection may be related to anaemia and haemoglobin level in the Chinese population.
